# Analysis of Risk Factors with Assessment of the Impact of the Microbiome on the Risk of Squamous Cell Carcinoma of the Larynx

**DOI:** 10.3390/jcm13206101

**Published:** 2024-10-13

**Authors:** Karolina Dorobisz, Tadeusz Dorobisz, Katarzyna Pazdro-Zastawny

**Affiliations:** 1 Department of Otolaryngology, Head and Neck Surgery, Wrocław Medical University, Borowska 213, 50-556 Wrocław, Poland; 2 Department of Vascular and General Surgery, Wrocław Medical University, Borowska 213, 50-556 Wrocław, Poland

**Keywords:** microbiome, laryngeal cancer, head and neck cancer, risk factors, dysbiosis

## Abstract

**Introduction**: Head and neck squamous cell carcinoma (HNSCC) ranks sixth among cancers in the world, and the 5-year survival rate ranges from 25% to 60%. The risk factors for HNSCC are primarily smoking, alcohol consumption and human papillomavirus (HPV). Data indicate that 15–20% of cancers are caused by infectious agents, 20–30% by smoking and 30–35% by unhealthy lifestyles, diet, lack of physical activity and obesity. Dysbiosis is a microbiome imbalance, which promotes oncogenesis by intensifying inflammatory processes and affecting the host’s metabolism. Profiling the microbiome in various types of cancer is currently the subject of research and analysis. However, there is still little information on the correlation of the microbiome with HNSCC and its impact on oncogenesis, the course of the disease and its treatment. **Objective**: The aim of the study was to prospectively assess risk factors with assessment of the impact of the microbiome on the risk of squamous cell carcinoma of the larynx. The study included a group of 44 patients diagnosed with squamous cell carcinoma of the larynx and 30 patients from the control group. **Results**: In the control group, bacteria of the normal microbiome dominated—the genus *Streptococcus*, *Gemella*, *Neisseria* and *Kingella*. In the group of patients with laryngeal cancer, *Prevotella*, *Clostridiales* and *Stomatobaculum* were found significantly more often. *Porphyromonas*, *Fusobacterium*, *Lactobacillus*, *Actinobacteria*, *Actinomyces* and *Shaalia odontolytica* were also found at a higher percentage in the study group. Analyzing the phylum, Firmicutes dominated in the control group; there were statistically significantly more of them than in patients from the study group. Bacteroides and Bacillota were found significantly more often in patients with laryngeal cancer. **Conclusions**: The importance of the microbiome in oncology has been confirmed in many studies. Independent risk factors for laryngeal cancer were primarily a lower number of Firmicutes in the microbiome, but also an increased leukocyte level above 6.52 × 10^3^/mm and a decreased total protein level below 6.9 g/dL. *Prevotella*, *Clostridiales*, *Stomatobaculum*, *Porphyromonas*, *Fusobacterium*, *Lactobacillus*, *Actinobacteria*, *Actinomyces* and *Shaalia* were considered to be the bacteria contributing to the development of laryngeal cancer. *Streptococcus*, *Gemella*, *Neisserie* and *Kingella* were considered to be protective bacteria. Moreover, the study confirmed the significant impact of smoking, alcohol consumption and poor oral hygiene on the development of laryngeal cancer. The microbiome, its identification and manipulation may constitute a breakthrough discovery for improving the diagnosis and oncological therapy of laryngeal cancer, and also of the entire group of HNSCC. Profiling the microbiome may allow for personalized therapy related to its modification. Assessing the microbiome of patients diagnosed with cancer may provide an opportunity to predict treatment response and effectiveness.

## 1. Introduction

Head and neck squamous cell carcinoma (HNSCC) ranks sixth among the world’s most common cancers, registering over half a million new cases worldwide each year, and the 5-year survival rate ranges from 25% to 60% [1,2]. The risk factors for HNSCC are smoking, alcohol consumption and human papillomavirus (HPV) [3]. Data indicate that 15–20% of cancers are caused by infectious agents, 20–30% by smoking and 30–35% by unhealthy lifestyles, diet, lack of physical activity and obesity [4]. Oncogenic viruses have been identified as representatives of infectious agents; their action is primarily based on integration into the host genome and inactivation of tumor suppressor genes such as p53 in the case of HPV infection [5,6]. In the treatment of HNSCC, combined therapies are used, consisting of surgery, radiotherapy, chemotherapy and immunotherapy, but the effects of this treatment are not satisfactory and the survival rate of patients is low [7].

Next generation sequencing (NGS) has allowed us to change our view of the bacterial world. The microbiome consists of the genome of the microbiota, as well as products of the host’s microflora such as plasmid DNA, viruses, fungi and archaea [8]. The human microbiome is individually variable, and its composition may be primarily influenced by environmental factors and the host [9,10]. Many studies are currently analyzing the functions of the microbiome in the pathogenesis of many diseases, and its relationship with inflammatory bowel diseases, obesity, allergy, autism, depression and cancer has been proven [11,12,13]. Dysbiosis is a microbiome imbalance, promotes oncogenesis by intensifying inflammatory processes and affecting the host’s metabolism. The microbiome plays a very important role in the functioning of the immune system [14,15]. Profiling the microbiome in various types of cancer is currently the subject of research and analysis. However, there is still little information on the correlation of the microbiome with HNSCC and its impact on oncogenesis, the course of the disease and its treatment. New treatment options that may be provided by expanding knowledge on this subject are very important for improving the treatment outcomes of patients with laryngeal cancer.

## 2. Materials and Methods

### Study Group

The study included a group of 44 patients diagnosed with squamous cell carcinoma of the larynx, aged from 43 to 73 years (mean *M* = 63.4, *SD* = 9.0). The study was conducted at the Department of Otolaryngology, Head and Neck Surgery of the University Clinical Hospital in Wrocław. Patients were included in the study consecutively. The study was conducted in 2022 and 2023. Men constituted the majority of the study group (79.6%). The inclusion criteria for the project included patients with squamous cell carcinoma of the larynx before undergoing oncological treatment. The exclusion criteria for the project included patients with chronic inflammation of the upper and lower respiratory tract, had used antibiotics in the last 6 months, and had a history of other cancers and acute infections.

Each patient underwent diagnostic imaging, a biopsy was taken from the focal lesion, and TNM was determined, based on which the patient’s treatment was decided. The general condition of the patients was assessed according to the ECOG scale. Laboratory tests were analyzed in each patient; blood count and nutritional parameters—total protein, total cholesterol, HDL, LDL, iron level, coagulation system, TSH, CRP protein. The nutritional status of patients was assessed, qualifying patients as satisfactorily nourished, at risk of malnutrition, or with malnutrition. Then, a swab was taken from each patient for microbiological culture and a swab to assess the microbiome.

## 3. Microbiome Profiling Workflow

1. DNA isolation from cotton swab samples using a commercial kit following the manufacturer’s protocol (GeneMATRIX Swab-Extract DNA Purification Kit, Eurx, Gdańsk, Poland).
2. Quality control of isolated DNA—concentration and purity evaluation (Qubit 4 Fluorometer, Invitrogen and DeNovix DS-11 spectrophotometer, Connecticut, US); DNA integrity check by electrophoresis on 1.5% agarose gel.
3. Amplicon libraries construction following two rounds of PCR amplification-
  - Amplification of specific target DNA region of bacterial 16S ribosomal RNA (V3–V4) using universal primers connected with Illumina sequencing adapters; PCR Clean-Up using AMPure XP beads, Indianapolis, US.
  - Index PCR attaching dual indices and Illumina sequencing adapters using the Nextera XT Index Kit. San Diego, US; PCR Clean-Up using AMPure XP beads, Indianapolis, US.
4. Library QC, quantification, normalization, and pooling.
5. Sequencing on MiSeq–Using paired 300-bp reads.

### Control Group

The control group comprised 30 healthy patients aged 45 to 73 years (mean *M* = 61.4, *SD* = 8.2). People classified in this group did not suffer from cancer. Exclusion criteria included the same conditions as reported in the study group.

The study was approved by the bioethical committee of Wroclaw Medical University, Poland, 150/22 in 2022. The study was conducted in accordance with the Declaration of Helsinki, and all participants were informed about the purpose of the study and gave their written consent.

## 4. Statistical Analysis

The STATISTICA v. 13.3 program (TIBCO Sotfware Inc., Palo Alto, CA, USA) and a Microsoft Excel (Microsoft Office LTSC Standard 2021) spreadsheet were used to conduct statistical analysis of the results of clinical trials and surveys.

The assessment of compliance of the empirical distributions of continuous quantitative variables with theoretical normal distributions was checked using the Shapiro-Wilks test. For quantitative variables, mean (M), standard deviation (SD), median (Me), lower (Q1) and upper quartile (Q3) values were calculated. In the tables and figures, variables with a distribution close to normal were determined by mean and standard deviation—*M* ± *SD*, variables with a distribution significantly different from normal were presented as medians and quartiles—*Me* [*Q*1; *Q*3]. For qualitative (nominal, e.g., gender, marital status, etc.) and ordinal (e.g., education level, nutritional status, etc.) variables, frequencies (*n*) and percentages (%) were calculated and collected in contingency tables.

Hypotheses about the lack of a relationship between two qualitative characteristics were verified using the Pearson Chi-square test or Fisher’s exact test. The significance of differences in mean values of variables with a distribution close to normal and with homogeneous variances in two groups was checked using the Student’s *t*-test. Homogeneity of variances was checked using the Brown-Forsyth test and Levene’s test. The significance of differences in mean values of variables with a significantly non-normal distribution or with heterogeneous variances in two groups was checked using the Mann-Whitney *U* test. In the case of a larger number of groups, the Kruskal-Wallis test was used.

The cut-off values of continuous variables separating two conditions (e.g., presence of cancer) based on parameters of laboratory tests, questionnaires, interviews and microbiome were determined based on ROC curve analysis and Youden index. The area under the curve (AUC) was estimated for each prognostic parameter, and the sensitivity, specificity and accuracy of the test were calculated for the proposed cut-off value. Multivariate logistic regression analysis was used to determine the independent factors associated with the occurrence of head and neck cancers. The dichotomous dependent variable was the diagnosis of the disease (Examined group = 1, Control group = 0), and the independent variables were clinical, interview and biometric parameters. Logit models have been proposed to estimate the probability of head and neck cancer occurrence. In all statistical tests used, the significance level was α = 0.05. The result of a statistical test was considered significant when the test probability was *p* < 0.05.

## 5. Results

### 5.1. Analysis of General Characteristics of Patients in the Study and Control Group

In the study group, statistically significantly more patients had lower education and were more often single, not in a partnership and without family support. Both groups did not differ in terms of age, gender, place of residence, and body mass index (*p* < 0.05). These data are presented in Table 1.

### 5.2. Analysis of Medical History of the Control Group and the Study Group

Assessment of patients’ performance differed significantly in the study group and in the control group. In the study group, 32 (72.73%) patients were classified as ECOG stage one or stage two, while in the control group, only eight (26.7%) patients were classified as stage one, and the remaining patients were assessed as normal (grade 0). Patients with laryngeal cancer reported swallowing disorders significantly more often, and this occurred in 36.4% of patients. Patients from the study group smoked tobacco and regularly consumed alcohol more often than the control group - this concerned 97.7% and 54.5% of patients with laryngeal cancer, respectively, the difference was statistically significant. In the control group, 60% of patients were smokers, and none consumed alcohol regularly. Statistically, patients in the study group had periodontal disease more often—this problem affected a total of 81.2% of patients in this group compared to 10% in the control group. The nutritional status of patients with laryngeal cancer was significantly worse than that of patients from the control group. Patients from the study group had signs of malnutrition; it was found in 59.1%, while the risk of malnutrition was 22.7%. In the control group, all patients were properly nourished. These data are presented in Table 2.

### 5.3. Characteristics of the Study Group

In the study group, 75% were diagnosed with glottis cancer and 25% with epiglottis cancer. The tumor was described as T1 in 25% of patients, T2 in 38.7%, T3 in 29.5%, while 6.8% of patients were stage T4a. The absence of lymph node metastases was found in 56.8% of patients, the N1 feature occurred in 11.3% of patients, N2a-c was found in a total of 29.6% of patients, and N3a in 2.3%. No distant metastases were found in any patient from the study group. All patients were treated radically. A total of 25.5% of patients were in stage I, 20.4% in stage II, 22.8% in stage III, 11.4% in stage IVa, and 20.4% in stage IVb. Surgical treatment was used in 45.5% of patients, radiotherapy in 90.9%, and chemotherapy in 38.6%. Surgical treatment only was performed in 6.8% of patients, surgical treatment with adjuvant radiotherapy in 18.2%, radical radiotherapy in 47.8% and surgical treatment with adjuvant radiochemotherapyin 13.6% of patients. These data are presented in Table 3.

### 5.4. Analysis of Laboratory Parameters between the Study Group and the Control Group

Patients in the study group had statistically significantly lower hemoglobin levels and increased leukocyte levels. The APTT time was prolonged compared to the control group, but it was within the normal range. Similarly, the INR level was statistically significantly higher in the study group compared to the control group. The total protein level was significantly reduced in the group of patients with laryngeal cancer compared to patients from the control group. CRP protein was significantly elevated compared to the control group and slightly exceeded the accepted norm (range 5.9–25.9). The levels of total cholesterol, LDL, HDL and triglycerides were significantly reduced in the study group, as well as iron levels. The TSH level in the study group was within the normal range, but was statistically significantly lower than the level of the control group. These results are presented in Table 4.

### 5.5. Analysis of Bacterial Cultures between the Study Group and the Control Group

In the study of classic cultures, statistically significantly more results with the physiological flora of *Streptococcus oralis* were obtained in patients from the control group; this bacterium was found much less frequently in the study group. *Candida albicans* was found more often in the study group than in the control group, but the difference was not significant. In the study group, pathogenic bacteria such as *Staphylococcus aureus*, *Pseudomonas*, *Serratia mercescens*, *Bifidobacterium*, *Corynebacterium*, *Enterococcus*, *Klebsielle*, *Enterobacter*, *Serratia*, *Lacticaseibicillus*, *Morganelle*, *Proteus*, *Veillonelle*, *Escherichie* were found; these bacteria were not cultured in any person from the control group. Culture results marked as sterile were obtained in 30% of patients from the control group and in 22.7% of patients from the study group. These data are presented in Table 5.

### 5.6. Microbiome Analysis

In the group of patients with laryngeal cancer, a lower percentage of *Streptococcus* (7.4% vs. 29.6%) and *Gemella* (0.4% vs. 1.7%) was found compared to the control group, and these differences were statistically significant. In the group of patients with laryngeal cancer, *Prevotella* dominated in microbiome profiling (total 28.9% vs. 14.2%) compared to the control group, and this difference was statistically significant. *Clostridiales* and *Stomatobaculum longum* also occurred significantly more often in the group of patients with laryngeal cancer. In the study group, *Porphyromonas*, *Fusobacterium*, *Lactobacillus*, *Actinobacteria*, *Actinomyces* and *Shaalia odontolytica* were also found in higher percentages, but these differences were not statistically significant. *Neisseria*, *Kingella* and *Rothia* predominated in the control group compared to the study group, but these differences were not statistically significant. These results are presented in Table 6.

Table 7 shows bacteria detected only in the microbiome analysis in the study group; they were not present in any of the people in the control group. Statistically significant differences were observed with *Oribacteria*, *Bergeyella cardium*, *Catonella*, *Eubacterium* and *Peptostreptococcus.*

### 5.7. Microbiome Analysis in Terms of Phylum

**Figure 1:**
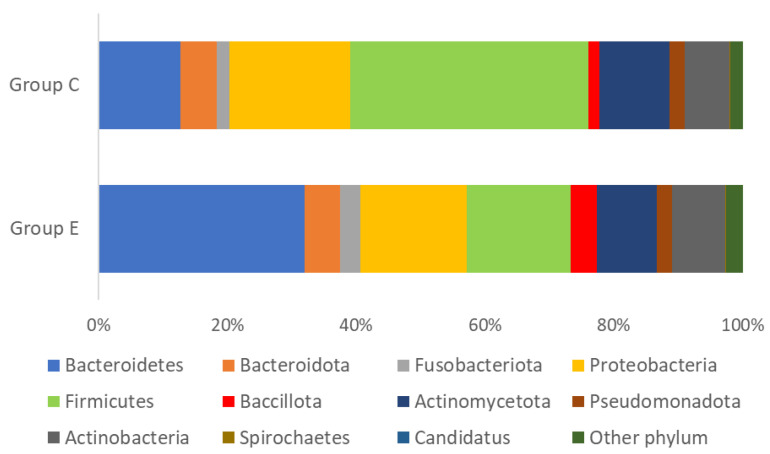
Microbiome of the study and control groups.

In the group of patients from the study group, bacteria from the *Bacteroides* and *Bacillota* groups predominated, and the differences compared to the control group were statistically significant. The control group was dominated by *Firmicutes* and this difference was statistically significant. These data are presented in Table 8 and Figure 1.

For a *Bacteroidetes* cut-off value of ≥24.7%, the test sensitivity is Sens = 72.7%, specificity Spec = 86.7% and likelihood ratio LR(+) = 5.46. For a *Firmicutes* cutoff of ≤22.1%, the test sensitivity is Sens = 84.1%, specificity Spec = 83.3% and likelihood ratio LR(+) = 5.05. For a *Bacillota* cut-off value of ≥1.7%, the test sensitivity is Sens = 65.9%, specificity Spec = 80.0% and likelihood ratio LR(+) = 3.30.

Multivariate logistic regression analysis was used to determine the independent factors associated with the occurrence of head and neck cancers. The dichotomous dependent variable was the diagnosis of the disease (group E = 1, group C = 0), and the independent variables were clinical parameters. Cut-off values for continuous variables were determined based on ROC curve analysis.

Independent predictors of laryngeal cancer were a leukocyte count of at least 6.52 × 10^3^/mm, total protein level below 6.9 g/dL and the share of bacteria from the Firmicutes phylum in the microbiome below 22.1% (Table 9).

The model enabling the estimation of the probability of head and neck cancer occurrence takes the logit form:

Pr{cancer = 1|X} = logit(−6.71 + 4.32 × (Leukocytes < 6.52 × 10^3^/mm) +

               + 3.66 × (Total protein < 6.9 g/dL) + 4.30 × (Firmicutes < 22.1%)

**Figure 2:**
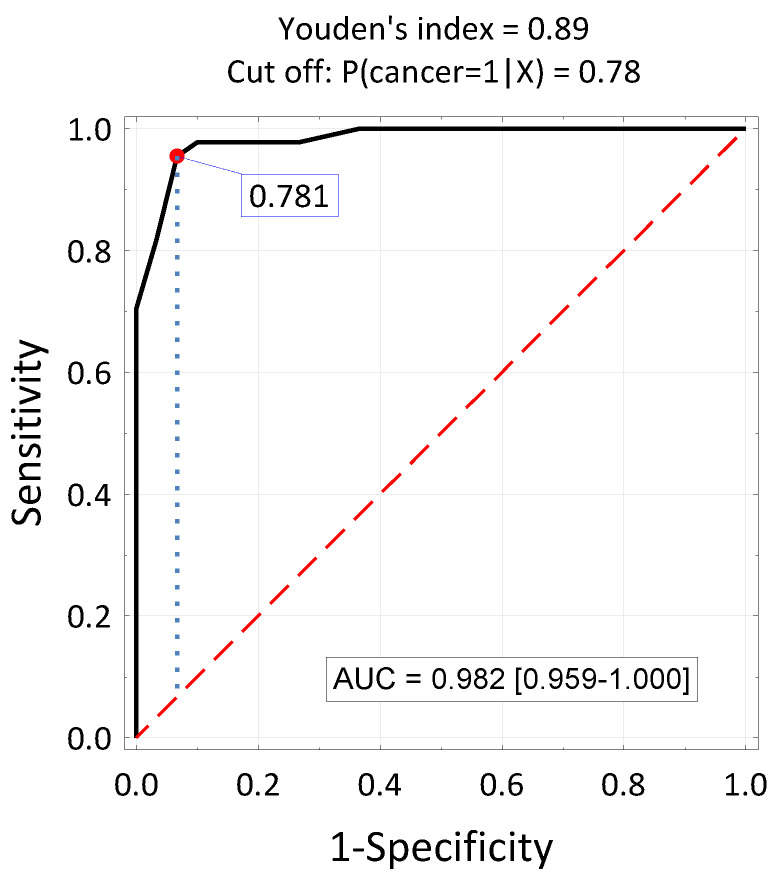
ROC curve for estimating the probability of the presence of head and neck cancer based on a logit model taking into account the number of leukocytes, total protein concentration and the percentage of bacteria from the Firmicutes phylum in the microbiota. Cut-off value and area under the curve.

For the cut-off value calculated from the logit model Pr(cancer = 1|x) = 0.781, the test sensitivity is Sens. = 95.5%, specificity Spec. = 93.3%, Accuracy Acc. = 94.6%, and the positive log likelihood LR(+) = 14.3- Figure 2.

## 6. Discussion

HNSCC is a group of cancers with a 5-year mortality rate of 40%. Despite many activities aimed at preventing smoking, alcohol consumption and HPV infections, the effects of improving prognosis are still unsatisfactory, and survival rates in the case of HNSCC have not improved over the last many years. New methods of prevention, early detection and treatment of HNSCC are needed. Additionally, an important factor influencing mortality is the high level of disease recurrence after treatment. It seems necessary to develop new procedures and try to find additional risk factors responsible for the development of HNSCC. The microbiome plays an important role in oncogenesis, the course and effectiveness of cancer treatment [16,17]. The microbiome influences various cancers through inflammatory reactions; enzymes, endotoxins and bacterial metabolic products can damage cell DNA and influence metabolism, inducing tumor suppressor genes and promoting proto-oncogenes [18]. In HNSCC, it is important for bacteria to activate pro-oncogenic substances such as acetaldehyde, a metabolite of ethyl alcohol [19]. The impact of the microbiome on the development of cancer may prove crucial for improving the effectiveness of prevention, diagnosis and treatment, but there are few publications discussing this topic. Inflammation is very important in the process of cancer development and is responsible for angiogenesis, the rate of tumor growth and metastasis. Uncontrolled inflammation leads to cell hypoxia and their necrosis, which facilitates the growth of anaerobic bacteria [20]. Dysbiosis is a microbiome imbalance, which affects the local and systemic immune response. Dysbiosis has been confirmed in the pathogenesis of many cancers [21,22,23,24,25]. It has also been shown that microbiome disorders affect the effectiveness of treatment, especially radiotherapy and immunotherapy [26,27]. The microbiome is modified by external factors such as diet, medications, smoking or alcohol consumption; its composition also depends on the immune system and the genetic susceptibility of the host [28]. The gastrointestinal microbiome as the richest system and its impact on many diseases are the topic of many studies. The microbiome of the upper respiratory tract, and the disruption of immune homeostasis by dysbiosis, also significantly affects many diseases locally and systemically. The first reports on the influence of bacteria on the development of HNSCC showed *Fusobacterium nucleatum* and *Prophyromonaxs gingivalis* in tumor tissues in culture tests [29]. Interest in the world of oncogenic bacteria in relation to the widely known group of oncogenic viruses gave rise to research on this topic.

Research on the microbiome uses 16S rRNA sequencing with analysis of taxonomic distribution and assessment of microbiome diversity, and this method was also used in the discussed work. The discussed study presents significant differences in the microbiome in patients with squamous cell carcinoma of the larynx compared to patients from the control group. In the group of healthy patients, bacteria of the normal microbiome predominated—the genus *Streptococcus*, *Gemella*, *Neisseria* and *Kingella*. In the group of patients with HNC, *Prevotelle*, *Clostridiales* and *Stomatobaculum* were found significantly more often. *Porphyromonas*, *Fusobacterium*, *Lactobacillus*, *Actinobacteria*, *Actinomyces* and *Shaalia odontolytica* were also found in a higher percentage in the HNC group. Rare bacteria were frequently found in the HNC microbiome. Analyzing the phylum, *Firmicutes* bacteria dominated in the control group, and there were statistically significantly more of them than in patients from the study group. The *Bacteroides* and *Bacillota* phyla were found significantly more often in patients with HNC. Stashenko et al. [30] used a mouse model to prove the influence of the microbiome on the course of cancer; mice with the transferred microbiome from tongue cancer had a significant increase in the number of tumors and their size. This may suggest a risk of faster progression, worse response to treatment and risk of disease recurrence in patients with severe dysbiosis. Frank et al. [31] also showed that the salivary microbiome differs significantly between patients with HNSCC and the control group. An increased amount of *Lactobacillus* and a decreased amount of Neisseria was found in the saliva of patients with HNSCC compared to controls, and similar results were also obtained in the discussed study. Frank et al. [31] also assessed the effect of antibiotic therapy on the progression of cancer in a mouse model; a reduction in tumor mass was demonstrated in mice treated with antibiotic therapy. In their study, Gong et al. [32] showed a significant reduction of *Streptococcus* from 56% in the microbiome in the control group to 25% in the study group. In this study, *Fusobacterium* and *Prevotella* weresignificantly more in laryngeal cancer [32]; the results of these authors also correlate with the results of the discussed work. In another study, Hayes et al. [33] showed that an increased amount of *Corynebacterium*, *Kingella*, *Neisseria* and *Streptococcus* was associated with a reduced risk of laryngeal cancer. In a study by Hayes et al. [33], bacteria associated with periodontal disease and caries, such as *Porphyromonas gingivalis*, *Tannerella forsythia, Aggregatibacter*, and *Streptococcus mutans* were not associated with the risk of HNSCC. However, in patients with poor oral hygiene, dysbiosis causes inflammation, but it seems that it is not the bacteria of periodontal disease that are the risk factors, but the reduction of the amount of normal bacterial flora by pathological strains. Such observations were also made in the current work. *Porphyromonas gingivalis* has the ability to stimulate oncogenesis in the oral cavity [34]; high levels of class G antibodies against this bacterium in serum found in patients with gastrointestinal cancer and HNSCC are associated with higher mortality [35]. An important risk factor for laryngeal cancer seems to be a reduction in the number of *Streptococcus*, *Gemella* and *Kingella* bacteria and a simultaneous increase in the number of bacteria causing dysbiosis. Many studies point to various bacteria that may have a significant impact on the development of cancer. However, it can be concluded that the reduction of bacteria normally living in the upper respiratory tract may be the most important element causing dysbiosis. In the study by Hayes et al. [33], after excluding other risk factors such as smoking, alcohol consumption or HPV infection, they confirmed that the composition of the microbiome did not change, which supports the independent influence of dysbiosis on HNSCC. However, other authors have shown that smokers, regardless of alcohol consumption, showed lower species richness, including reductions in *Neisseria*, *Gemella* and *Peptostreptococcus* [36]. Lafuente Ibáñez de Mendoza et al. [37] in a recently published work presented the results of the assessment of the microbiome in oral cancer and precancerous conditions, finding dysbiosis in these conditions, but the loss of diversity was observed only in patients with diagnosed cancer. The discussed study also analyzed the systemic inflammation of the body in patients with locoregional cancer, and statistically significantly higher levels of leukocytes and CRP were confirmed compared to the control group. Chronic inflammation caused by persistent bacterial infection induces carcinogenesis [38]. The expression of pro-inflammatory cytokines IL-1 in periodontal diseases has been associated with microbial-induced carcinogenesis [39]. Similar observations regarding inflammation have also been observed by other researchers [40,41,42]. The inflammation is accompanied by malnutrition in patients with reduced levels of total protein, as shown in the discussed work. Malnutrition with reduced total protein levels in the low stages of cancer and occurring before diagnosis shows that it is also an important risk factor for HNSCC, and the relationship between malnutrition and prognosis after oncological surgery was also noticed by other authors [43,44].

## 7. Limitations of the Study

It is worth conducting a large clinical study assessing the impact of the microbiome on the risk of developing squamous cell carcinoma of the head and neck, taking into account the impact of other risk factors and modifications of the microbiome with various groups of antibiotics or probiotics. Further research and observations along with attempts to modify the microbiome are needed to obtain better results of HNC treatment.

## 8. Conclusions

The importance of the microbiome in oncology has been confirmed in many studies. The independent risk factors for laryngeal cancer were primarily a lower number of *Firmicutes* in the microbiome, but also an increase in the leucocyte level above 6.52 × 10^3^/mm and a decrease in total protein below 6.9 g/dL. *Prevotella*, *Clostridiales*, *Stomatobaculum*, *Porphyromonas*, *Fusobacterium*, *Lactobacillus*, *Actinobacteria*, *Actinomyces* and *Shaalia* were considered to be the bacteria contributing to the development of laryngeal cancer. Protective bacteria were considered to be *Streptococcus*, *Gemella*, *Neisserie* and *Kingella*. Moreover, the study confirmed the significant impact of smoking, alcohol consumption, and poor oral hygiene on the development of laryngeal cancer. The microbiome, its identification and manipulation may be a breakthrough discovery in addition to the diagnosis and oncological therapy of laryngeal cancer, and also of the entire group of HNC. It may allow for personalized therapy related to microbiome modification. Assessment of the microbiome of patients diagnosed with cancer may provide the opportunity to predict the response to treatment and its effectiveness.

## Figures and Tables

**Table 1:** General characteristics of patients in the study and control groups and the results of significance and independence tests.

Variable	Exam Group (E) N = 44	Control Group (C) N = 30	E Vs. C *p*-Value	Post-Hoc Test
Gender:			0.202 ^a^	
Male, *n* (%)	35 (79.6%)	19 (63.3)		
Female, *n* (%)	9 (20.4%)	11 (36.7%)		
Age (years):			0.703 ^b^	
*M* ± *SD*	63.4 ± 9.0	62.6 ± 6.8		
Education:			0.001 ^a^	
Primary	14 (31.8%)	1 (3.3%)		0.003
Secondary	25 (56.8%)	16 (53.4%)		0.773
Incomplete higher	1 (2.3%)	6 (20.0%)		0.011
Higher	4 (9.1%)	7 (23.3%)		0.092
Place of residence:			0.770 ^a^	
Village	11 (25.0%)	8 (26.7%)		
Town up to 20,000	6 (13.6%)	2 (6.7%)		
21–50,000 inhabitants	7 (15.9%)	4 (13.3%)		
Over 50,000	20 (45.5%)	16 (53.3%)		
Economic zone/urban area:			0.871 ^a^	
Yes	26 (59.1%)	18 (60.0%)		
No	18 (40.9%)	12 (40.0%)		
Marital status:			0.004 ^a^	
Single	17 (38.6%)	3 (10.0%)		0.007
Partner/married relationship	17 (38.6%)	23 (76.7%)		0.001
With family support	10 (22.8%)	4 (13.3%)		0.306
BMI (kg/m^2^):			0.100 ^c^	
*Me [Q*1*–Q*3*]*	22.7 [21.3–24.2]	23.4 [22.4–25.1]		

*n*—number, (%)—percentile, *M*—mean, *SD*—standard deviation, *Me*—median, *Q*1–*Q*3—lower and upper quartile, *p*—test significance level, ^a^—Pearson’s chi-square test, ^b^—Student’s *t* test, ^c^—Mann-Whitney *U* test.

**Table 2:** Number (percentage) of people differing in the presence of cancer and analyzed clinical features, as well as the results of independence and significance tests.

Variable	Group E N = 44	Group C N = 30	E vs. C *p*-Value	Post-Hoc Test
ECOG scale (score):			<0.001	
0—asymptomatic, *n* (%)	12 (35.3%)	22 (64.7%)		0.013
1—symptomatic, but completely ambulatory	30 (78.9%)	8 (21.1%)		<0.001
2—Symptomatic, <50% in bed during the day	2 (100.0%)	0 (0.0%)		<0.001
Swallowing disorders (yes)	16 (36.4%)	0 (0.0%)	<0.001	
Smoking	43 (97.7%)	18 (60.0%)	<0.001	
Drinking alcohol regularly	24 (54.5%)	0 (0.0%)	<0.001	
Dental condition:			<0.001	
1—Normal	8 (18.2%)	30 (100.0%)		<0.001
2—Cavities, caries, periodontal disease	30 (68.2%)	0 (0.0%)		<0.001
3—Edentulism	6 (13.6%)	0 (0.0%)		0.035
Nutritional status:			<0.001	
1—Satisfactory	8 (18.2%)	30 (100.0%)		<0.001
2—Risk of malnutrition	10 (22.7%)	0 (0.0%)		0.005
3—Malnutrition	26 (59.1%)	0 (0.0%)		<0.001

**Table 3:** Clinical characteristics of patients with laryngeal cancer.

Study Group	N = 44 (100%)
Tumor location:	
Glottis	33 (75.0%)
Epiglottis	11 (25.0%)
Tumor:	
T1	11 (25%)
T2	17 (38.7%)
T3	13 (29.5%)
T4a	3 (6.8%)
Node:	
N0	25 (56.8%)
N1	5 (11.3%)
N2a	1 (2.3%)
N2b	7 (15.9%)
N2c	5 (11.4%)
N3a	1 (2.3%)
M0	44 (100.0%)
Stage:	
AND	11 (25.0%)
II	9 (20.4%)
III	10 (22.8%)
Iva	5 (11.4%)
IVb	9 (20.4%)
Treatment *:	
Surgery	20 (45.5%)
Radiotherapy	40 (90.9%)
Chemotherapy	17 (38.6%)
Only surgery	3 (6.8%)
Surgery + radiotherapy	8 (18.2%)
Only radiotherapy	21 (47.8%)
Surgery + radiotherapy + chemotherapy	6 (13.6%)
Radiotherapy + chemotherapy	6 (13.6%)

* Multiple choice question, percentages do not add up to 100.

**Table 4:** Clinical characteristics of patients in the study group.

Variable, *Me [Q*1*–Q*3*]*	Study Group (S) N = 44	Control Group (C) N = 30	S vs. C *p*-Value
Hemoglobin (g/dL)	12.3 [11.1–13.3]	13.5 (12.8–14.6)	<0.001
Leukocytes (×10^3^/mm)	9.1 [8.1–11.3]	5.1 [4.3–6.3]	<0.001
APTT(s)	28.5 [26.3–31.7]	27.5 [26.0–29.3]	0.050
INR (-)	1.05 [0.98–1.15]	1.00 [0.96–1.04]	0.007
Total protein (g/dL)	6.3 [5.9–6.8]	7.3 [7.1–7.8]	<0.001
CRP (mg/L)	12.0 [6.3–25.9]	3.2 [2.1–4.1]	<0.001
Total cholesterol [mg/dL]	144 [112–168]	184 [175–192]	<0.001
LDL cholesterol [mg/dL]	105 [66–125]	124 [115–135]	<0.001
HDL cholesterol [mg/dL]	34 [27–41]	45 [34–48]	<0.001
Triglycerides [mg/dL]	109 [98–135]	128 [117–140]	0.009
Iron level (mcg/dL)	45 [32–57]	75 [73–87]	<0.001
TSH (mIU/L)	1.42 [1.03–2.22]	2.56 [1.98–2.98]	<0.001

**Table 5:** Number (percentage) of people differing in the presence of cancer and isolated types of bacteria and fungi, as well as in the results of independence tests (Fisher’s exact test).

Cultureresult	Group S N = 44	Group C N = 30	S vs. C *p*-Value
*Streptococcus oralis*	16 (36.4%)	21 (70.0%)	0.009
*Staphylococcus aureus*	4 (9.1%)	0 (0.0%)	0.142
*Streptococcus pneumoniae*, *pneumococcus*	0 (0.0%)	0 (0.0%)	1.00
*Candida albicans*	17 (38.6%)	5 (16.7%)	0.069
*Neisseria*	4 (9.1%)	4 (13.3%)	0.707
*Pseudomonas*	5 (11.4%)	0 (0.0%)	0.076
*Serratia mercescens*	3 (6.8%)	0 (0.0%)	0.276
*Bifidobacterium longum*	2 (4.6%)	0 (0.0%)	0.511
*Corynebacterium*	1 (2.3%)	0 (0.0%)	1.000
*Enterococcus faecalis*	1 (2.3%)	0 (0.0%)	1.000
*Klebsiella*, *Enterobacter* and *Serratia*	3 (6.8%)	0 (0.0%)	0.276
*Citrobacter freundii*	1 (2.3%)	0 (0.0%)	1.000
*Lacticaseibicillus paracasei*	2 (4.6%)	0 (0.0%)	0.511
*Morganella morganii*	2 (4.6%)	0 (0.0%)	0.511
*Streptococcus dysgalactiae*	1 (2.3%)	0 (0.0%)	1.000
*Proteus*	0 (0.0%)	0 (0.0%)	1.000
*Enterobacter cloacae*	0 (0.0%)	0 (0.0%)	1.000
*Veillonella parvula*	1 (2.3%)	0 (0.0%)	1.000
*Escherichia coli*	1 (2.3%)	0 (0.0%)	1.000
Absentee	10 (22.7%)	9 (30.0%)	0.590

**Table 6:** Descriptive statistics of the percentage of isolated bacteria and fungi in the study and control groups and the results of significance tests (Mann-Whitney U test).

Culture Result—Genus (%)	Group S	Group C	*p*-Value
*Streptococcus*	7.4 [4.4–10.2]	29.6 [20.0–36.3]	<0.001
*Prevotella melaninogenica*	13.2 [4.0–18.7]	5.5 [1.0–11.6]	0.005
*Prevotella*	15.7 [8.1–24.9]	5.0 [1.9–7.3]	<0.001
*Rothia micilaginosa*	3.2 [1.6–8.1]	9.2 [5.1–14.7]	0.004
*Aggregatibacter*	0.0 [0.0–0.3]	0.1 [0.0–0.4]	0.273
*Gemella*	0.4 [0.0–1.2]	1.7 [0.8–3.0]	<0.001
*Porphyromonas*	2.1 [0.3–5.7]	1.5 [0.0–4.9]	0.982
*Fusobacterium*	1.3 [0.1–2.7]	0.8 [0.3–2.1]	0.582
*Corynebacterium*	0.0 [0.0–0.5]	0.1 [0.0–2.0]	0.202
*Neisseria*	2.0 [0.2–6.8]	4.4 [0.0–10.7]	0.347
*Kingella*	0.0 [0.0–0.0]	0.0 [0.0–0.6]	0.316
*Lactobacillales*	2.7 [1.5–4.6]	2.6 [1.5–3.9]	0.451
*Actinobacteria*	1.9 [0.4–4.7]	1.5 [0.4–3.1]	0.447
*Actinomyces*	3.7 [1.0–6.6]	3.0 [1.4–5.6]	0.541
*Haemophilus*	4.0 [0.2–10.9]	6.6 [2.2–13.1]	0.102
*Capnocytophaga granulosa/gingivalis*	0.3 [0.0–1.5]	0.8 [0.0–2.8]	0.148
*Treponema*	0.0 [0.0–0.0]	0.0 [0.0–0.0]	0.947
*Candidadus Sacharimonas aalborgensis*	0.0 [0.0–0.0]	0.0 [0.0–0.0]	0.438
*Clostridiales*	0.5 [0.0–0.8]	0.0 [0.0–0.2]	0.004
*Veilonella*	0.0 [0.0–0.2]	0.0 [0.0–0.2]	0.688
*Campylobacter*	1.8 [0.5–3.7]	1.1 [0.0–2.1]	0.087
*Granulicatella*	0.0 [0.0–0.0]	0.0 [0.0–0.0]	0.425
*Cloacibacterium*	0.0 [0.0–0.0]	0.0 [0.0–0.0]	0.804
*Lautropia*	0.0 [0.0–1.2]	0.0 [0.0–1.0]	0.978
*Abiotrophia defective*	0.0 [0.0–0.0]	0.0 [0.0–0.0]	0.813
*Eikenella corrodens*	0.0 [0.0–0.0]	0.0 [0.0–0.0]	0.406
*Shaalia odontolytica*	1.1 [0.0–2.7]	0.0 [0.0–1.6]	0.098
*Leptotricha*	0.7 [0.0–1.8]	0.2 [0.0–0.8]	0.074
*Stomatobaculum longum*	0.3 [0.0–0.5]	0.0 [0.0–0.0]	0.005
*Granucicatella elegans*	0.0 [0.0–0.0]	0.0 [0.0–0.0]	0.512
*Tannerell*	0.0 [0.0–0.3]	0.0 [0.0–0.0]	0.438
*Pasteurellaceae*	0.2 [0.0–0.7]	0.5 [0.0–1.2]	0.198
*Bifidobacteriaceae*	0.0 [0.0–0.0]	0.0 [0.0–0.0]	0.241
*Atopobium*	0.0 [0.0–0.0]	0.0 [0.0–0.0]	0.218

**Table 7:** Descriptive statistics of the percentage of isolated rare bacteria and fungi in the study and control groups and the results of significance tests (Mann-Whitney U test).

Culture Result—Genus (%)	Group S	Group C	*p*-Value
*Oribacterium*	0.0 [0.0–0.4]	0.0 [0.0–0.0]	0.005
*Enterobacteriaceae*	0.0 [0.0–0.0]	0.0 [0.0–0.0]	0.624
*Parascardovia denticolens*	0.0 [0.0–0.0]	0.0 [0.0–0.0]	0.745
*Butyrivibrio*	0.0 [0.0–0.0]	0.0 [0.0–0.0]	0.745
*Cardiobacterium hominis*	0.0 [0.0–0.0]	0.0 [0.0–0.0]	0.512
*Bergeyella cardium*	0.0 [0.0–0.2]	0.0 [0.0–0.0]	0.013
*Catonella*	0.0 [0.0–0.2]	0.0 [0.0–0.0]	0.048
*Vallitalae okinawensis*	0.0 [0.0–0.0]	0.0 [0.0–0.0]	0.624
*Olsenella*	0.0 [0.0–0.0]	0.0 [0.0–0.0]	0.512
*Mogibacterium*	0.0 [0.0–0.0]	0.0 [0.0–0.0]	0.250
*Eubacterium*	0.0 [0.0–0.2]	0.0 [0.0–0.0]	0.032
*Sulfurihydrogenibium*	0.0 [0.0–0.0]	0.0 [0.0–0.0]	0.324
*Oceanivirga miroungae*	0.0 [0.0–0.0]	0.0 [0.0–0.0]	0.873
*Peptostreptococcus anaerobius*	0.0 [0.0–0.5]	0.0 [0.0–0.0]	0.013
*Mycoplasma salivarius/faucium*	0.0 [0.0–0.0]	0.0 [0.0–0.0]	0.250
*Dialister pneumosintes*	0.0 [0.0–0.0]	0.0 [0.0–0.0]	0.512
*Pseudoramibacter alactolyticus*	0.0 [0.0–0.0]	0.0 [0.0–0.0]	0.873
*Slackia*	0.0 [0.0–0.0]	0.0 [0.0–0.0]	0.873
*Filifactor alocis*	0.0 [0.0–0.0]	0.0 [0.0–0.0]	0.250
*Gordonibacter*	0.0 [0.0–0.0]	0.0 [0.0–0.0]	0.873
*Lachnoanaerobaculum saburreum*	0.0 [0.0–0.0]	0.0 [0.0–0.0]	0.624
*Cryptobacterium curtum*	0.0 [0.0–0.0]	0.0 [0.0–0.0]	0.745
*Scardovia wiggsiae*	0.0 [0.0–0.0]	0.0 [0.0–0.0]	0.324
*Dysgonomonas*	0.0 [0.0–0.0]	0.0 [0.0–0.0]	0.512
*Parvimonas*	0.0 [0.0–0.0]	0.0 [0.0–0.0]	0.512
*Ihubacter*	0.0 [0.0–0.0]	0.0 [0.0–0.0]	0.624
*Phocaeicola abscessus*	0.0 [0.0–0.0]	0.0 [0.0–0.0]	0.745
*Marinifilum*	0.0 [0.0–0.0]	0.0 [0.0–0.0]	0.996
*Klebsiella*	0.0 [0.0–0.0]	0.0 [0.0–0.0]	0.873
*Staphylococcus*	0.0 [0.0–0.0]	0.0 [0.0–0.0]	0.745
*Anaerococcus*	0.0 [0.0–0.0]	0.0 [0.0–0.0]	0.873
*Eschericia coli*	0.0 [0.0–0.0]	0.0 [0.0–0.0]	0.873
*Salmonella*	0.0 [0.0–0.0]	0.0 [0.0–0.0]	0.873
*Shigella*	0.0 [0.0–0.0]	0.0 [0.0–0.0]	0.873
*Finegoldia*	0.0 [0.0–0.0]	0.0 [0.0–0.0]	0.996
*Lawsonella clevelandensis*	0.0 [0.0–0.0]	0.0 [0.0–0.0]	0.873
*Moryella indoligens*	0.0 [0.0–0.0]	0.0 [0.0–0.0]	0.745
*Bacillales*	0.0 [0.0–0.0]	0.0 [0.0–0.0]	0.996
Other genus	0.7 [0.3–2.8]	1.5 [0.7–3.1]	0.055

**Table 8:** Descriptive statistics of the percentage of identified bacteria and fungi in the study and control groups and results of significance tests (Mann-Whitney U test).

Culture Result—Phylum (%)	Group S	Group C	*p*-Value
*Bacteroidetes*	33.2 [21.2–44.6]	10.0 [4.2–18.9]	<0.001
*Bacteroidota*	3.2 [1.2–7.6]	3.8 [0.6–10.1]	0.830
*Fusobacteriota*	2.5 [0.3–4.2]	1.3 [0.5–3.2]	0.259
*Proteobacteria*	15.4 [5.9–21.4]	19.4 [9.6–27.2]	0.269
*Firmicutes*	13.9 [8.5–18.5]	35.9 [24.8–41.6]	<0.001
*Bacillota*	2.2 [0.7–4.9]	0.4 [0.2–1.6]	0.002
*Actinomycetota*	7.1 [4.0–12.3]	9.6 [5.5–17.5]	0.169
*Pseudomonadota*	0.7 [0.0–2.9]	1.5 [0.5–2.4]	0.226
*Actinobacteria*	6.7 [2.4–12.2]	5.4 [3.0–9.3]	0.509
Other phylum	0.7 [0.2–3.2]	1.4 [0.6–3.1]	0.127

**Table 9:** Results of univariate and multivariate logistic regression analysis.

	Univariate Analysis			Multivariate Analysis		
	B	Mr	OR	Beta	Mr	OR (95% CI)
Education ≤ 2	1.86	0.004	5.96	0	>0.05	1.00
Singles	1.735	0.013	5.67	0	>0.05	1.00
Hemoglobin ≤ 13.9	2.034	0.002	7.65	0	>0.05	1.00
Leukocytes ≥ 6.52	4.234	<0.001	69.0	4.319	0.006	75.1 (3.51–1608)
APTT ≥ 29.76	2.457	0.003	11.7	0	>0.05	1.00
INR ≥ 1.05	1.281	0.018	3.60	0	>0.05	1.00
Total protein ≤ 6.9	3.718	<0.001	41.2	3.663	0.007	39.0 (2.82–539)
CRP ≥ 4.76	5.213	<0.001	184	0	>0.05	1.00
Total cholesterol ≤ 167	2.708	<0.001	15.0	0	>0.05	1.00
LDL ≤ 110	2.472	0.001	11.8	0	>0.05	1.00
HDL ≤ 42	2.251	<0.001	9.50	0	>0.05	1.00
Triglycerides ≤ 113	2.072	0.001	7/94	0	>0.05	1.00
Iron level ≤ 65	5.254	<0.001	191	0	>0.05	1.00
TSH ≤ 1.73	2.959	<0.001	19.3	0	>0.05	1.00
ECOG ≥ 1	1.992	<0.001	7.33	0	>0.05	1.00
Smoking	3.356	0.003	28/7	0	>0.05	1.00
*Bacteroidetes* ≥ 24.7%	2.853	<0.001	17.3	0	>0.05	1.00
*Firmicutes* ≤ 22.1%	3.455	<0.001	31.7	4.303	0.005	74.0 (3.94–1388)
*Bacillota ≥* 1.7%	2.046	<0.001	7.73	0	>0.05	1.00

## Data Availability

The raw data supporting the conclusions of this article will be made available by the authors on request.

## References

[1] Bray F., Ferlay J., Soerjomataram I., Siegel R.L., Torre L.A., Jemal A. (2018). Global cancer statistics 2018: GLOBOCAN estimates of incidence and mortality worldwide for 36 cancers in 185 countries. CA Cancer J. Clin..

[2] Gatta G., Botta L., Sánchez M.J., Anderson L.A., Pierannunzio D., Licitra L. (2015). EUROCARE Working Group: Prognoses and improvement for head and neck cancers diagnosed in Europe in early 2000s: The EUROCARE-5 population-based study. Eur. J. Cancer.

[3] Parkin D.M., Bray F., Ferlay J., Pisani P. (2005). Global Cancer Statistics, 2002. CA Cancer J. Clin..

[4] Song Y., Li L., Ou Y., Gao Z., Li E., Li X., Zhang W., Wang J., Xu L., Zhou Y. (2014). Identification of genomic alterations in oesophageal squamous cell cancer. Nature.

[5] Huang T.T., Lai J.B., Du Y.L., Xu Y., Ruan L.M., Hu S.H. (2019). Current understanding of gut microbiota in mood disorders: An update of human studies. Front. Genet..

[6] Tornesello M.L., Annunziata C., Tornesello A.L., Buonaguro L., Buonaguro F.M. (2018). Human oncoviruses and p53 tumor suppressor pathway deregulation at the origin of human cancers. Cancers.

[7] Mesia R., Iglesias L., Lambea J., Martínez-Trufero J., Soria A., Taberna M., Trigo J., Chaves M., García-Castaño A., Cruz J. (2021). SEOM clinical guidelines for the treatment of head and neck cancer (2020). Clin. Transl. Oncol..

[8] Marchesi J.R., Ravel J. (2015). The vocabulary of microbiome research: A proposal. Microbiome.

[9] Dominguez-Bello M.G., Costello E.K., Contreras M., Magris M., Hidalgo G., Fierer N., Knight R. (2010). Delivery mode shapes the acquisition and structure of the initial microbiota across multiple body habitats in newborns. Proc. Natl. Acad. Sci. USA.

[10] Palmer C., Bik E.M., DiGiulio D.B., Relman D.A., Brown P.O. (2007). Development of the human infant intestinal microbiota. PLoS Biol..

[11] Forbes J.D., Van Domselaar G., Bernstein C.N. (2016). Microbiome survey of the inflamed and noninflamed gut at different compartments within the gastrointestinal tract of inflammatory bowel disease patients. Inflamm. Bowel Dis..

[12] Liu J., Liu C., Yue J. (2021). Radiotherapy and the gut microbiome: Facts and fiction. Radiat. Oncol..

[13] Rodriguez M., Wootla B., Anderson G. (2016). Multiple sclerosis, gut microbiota and permeability: Role of tryptophan catabolites, depression and the driving down of local melatonin. Curr. Pharm. Des..

[14] Mitsuhashi A., Okuma Y. (2018). Perspective on immune oncology with liquid biopsy, peripheral blood mononuclear cells, and microbiome with non-invasive biomarkers in cancer patients. Clin. Transl. Oncol..

[15] Floch P., Megraud F., Lehours P. (2017). Helicobacter pylori strains and gastric MALT lymphoma. Toxins.

[16] Baskar R., Dai J., Wenlong N., Yeo R., Yeoh K.W. (2014). Biological response of cancer cells to radiation treatment. Front. Mol. Biosci..

[17] Kareva I. (2019). Metabolism and gut microbiota in cancer immunoediting, CD8/Treg Ratios, immune cell homeostasis, and cancer (immuno) therapy: Concise review. Stem Cells.

[18] Irfan M., Delgado R.Z.R., Frias-Lopez J. (2020). The Oral Microbiome and Cancer. Front. Immunol..

[19] Hooper S.J., Wilson M.J., Crean S.J. (2009). Exploring the link between microorganisms and oral cancer: A systematic review of the literature. Head. Neck.

[20] Baban C.K., Cronin M., O’Hanlon D., O’Sullivan G.C., Tangney M. (2010). Bacteria as vectors for gene therapy of cancer. Bioeng. Bugs.

[21] Schwabe R.F., Jobin C. (2013). The microbiome and cancer. Nat. Rev. Cancer.

[22] Alfano M., Canducci F., Nebuloni M., Clementi M., Montorsi F., Salonia A. (2016). The interplay of extracellular matrix and microbiome in urothelial bladder cancer. Nat. Rev. Urol..

[23] Brennan C.A., Garrett W.S. (2016). Gut Microbiota, Inflammation, and Colorectal Cancer. Annu. Rev. Microbiol..

[24] Yu G., Gail M.H., Shi J., Klepac-Ceraj V., Paster B.J., Dye B.A., Wang G.Q., Wei W.Q., Fan J.H., Qiao Y.L. (2014). Association between upper digestive tract microbiota and cancer-predisposing states in the esophagus and stomach. Cancer Epidemiol. Biomark. Prev..

[25] Fan X., Alekseyenko A.V., Wu J., Peters B.A., Jacobs E.J., Gapstur S.M., Purdue M.P., Abnet C.C., Stolzenberg-Solomon R., Miller G. (2016). Human oral microbiome and prospective risk for pancreatic cancer: A population-based nested case-control study. Gut.

[26] Gopalakrishnan V., Spencer C.N., Nezi L., Reuben A., Andrews M.C., Karpinets T.V., Prieto P.A., Vicente D., Hoffman K., Wei S.C. (2018). Gut microbiome modulates response to anti-PD-1 immunotherapy in melanoma patients. Science.

[27] Dong J., Li Y., Xiao H., Zhang S., Wang B., Wang H., Li Y., Fan S., Cui M. (2021). Oral microbiota affects the efficacy and prognosis of radiotherapy for colorectal cancer in mouse models. Cell Rep..

[28] Miranda-Galvis M., Loveless R., Kowalski L.P., Teng Y. (2021). Impacts of Environmental Factors on Head and Neck Cancer Pathogenesis and Progression. Cells.

[29] Gholizadeh P., Eslami H., Yousefi M., Asgharzadeh M., Aghazadeh M., Kafil H.S. (2016). Role of oral microbiome on oral cancers, a review. Biomed. Pharmacother..

[30] Stashenko P., Yost S., Choi Y., Danciu T., Chen T., Yoganathan S., Kressirer C., Ruiz-Tourrella M., Das B., Kokaras A. (2019). The Oral Mouse Microbiome Promotes Tumorigenesis in Oral Squamous Cell Carcinoma. mSystems.

[31] Frank D.N., Qiu Y., Cao Y., Zhang S., Lu L., Kofonow J.M., Robertson C.E., Liu Y., Wang H., Levens C.L. (2022). A dysbiotic microbiome promotes head and neck squamous cell carcinoma. Oncogene.

[32] Gong H.L., Shi Y., Zhou L., Wu C.P., Cao P.Y., Tao L., Xu C., Hou D.S., Wang Y.Z. (2013). The Composition of Microbiome in Larynx and the Throat Biodiversity between Laryngeal Squamous Cell Carcinoma Patients and Control Population. PLoS ONE.

[33] Hayes R.B., Ahn J., Fan X., Peters B.A., Ma Y., Yang L., Agalliu I., Burk R.D., Ganly I., Purdue M.P. (2018). Association of Oral Microbiome With Risk for Incident Head and Neck Squamous Cell Cancer. JAMA Oncol..

[34] Katz J., Onate M.D., Pauley K.M., Bhattacharyya I., Cha S. (2011). Presence of Porphyromonas gingivalis in gingival squamous cell carcinoma. Int. J. Oral. Sci..

[35] Ahn J., Segers S., Hayes R.B. (2012). Periodontal disease, Porphyromonas gingivalis serum antibody levels and orodigestive cancer mortality. Carcinogenesis.

[36] Thomas A.M., Gleber-Netto F.O., Fernandes G.R., Amorim M., Barbosa L.F., Francisco A.L., de Andrade A.G., Setubal J.C., Kowalski L.P., Nunes D.N. (2014). Alcohol and tobacco consumption affects bacterial richness in oral cavity mucosa biofilms. BMC Microbiol..

[37] Lafuente Ibáñez de Mendoza I., Maritxalar Mendia X., García de la Fuente A.M., Quindós Andrés G., Aguirre Urizar J.M. (2020). Role of Porphyromonas gingivalis in oral squamous cell carcinoma development: A systematic review. J. Periodont Res..

[38] Hanahan D., Weinberg R.A. (2011). Hallmarks of cancer: The next generation. Cell.

[39] Kipanyula M.J., Seke Etet P.F., Vecchio L., Farahna M., Nukenine E.N., Nwabo Kamdje A.H. (2013). Signaling pathways bridging microbial-triggered inflammation and cancer. Cell Signal.

[40] Atasever Akkas E., Yucel B. (2021). Prognostic value of systemic ımmune ınflammation ındex in patients with laryngeal cancer. Eur. Arch. Otorhinolaryngol..

[41] Verro B., Saraniti C., Carlisi D., Chiesa-Estomba C., Maniaci A., Lechien J.R., Mayo M., Fakhry N., Lauricella M. (2023). Biomarkers in Laryngeal Squamous Cell Carcinoma: The Literature Review. Cancers.

[42] Mao Y., Fu Y., Gao Y., Yang A., Zhang Q. (2018). Platelet-to-lymphocyte ratio predicts long-term survival in laryngeal cancer. Eur. Arch. Otorhinolaryngol..

[43] Santos A., Santos I.C., Dos Reis P.F., Rodrigues V.D., Peres W.A.F. (2022). Impact of Nutritional Status on Survival in Head and Neck Cancer Patients After Total Laryngectomy. Nutr. Cancer.

[44] van Bokhorst-de van der Schueren M.A., van Leeuwen P.A., Sauerwein H.P., Kuik D.J., Snow G.B., Quak J.J. (1997). Assessment of malnutrition parameters in head and neck cancer and their relation to postoperative complications. Head. Neck.

